# Effects of transmission reduction by insecticide-treated bed nets (ITNs) on parasite genetics population structure: I. The genetic diversity of *Plasmodium falciparum *parasites by microsatellite markers in western Kenya

**DOI:** 10.1186/1475-2875-9-353

**Published:** 2010-12-06

**Authors:** Wangeci Gatei, Simon Kariuki, William Hawley, Feiko ter Kuile, Dianne Terlouw, Penelope Phillips-Howard, Bernard Nahlen, John Gimnig, Kim Lindblade, Edward Walker, Mary Hamel, Sara Crawford, John Williamson, Laurence Slutsker, Ya Ping Shi

**Affiliations:** 1 Malaria Branch, Division of Parasitic Diseases, Centers for Disease Control and Prevention, Atlanta, Georgia, USA; 2 Atlanta Research and Education Foundation, Atlanta, GA, USA; 3 Center for Global Health Research, Kenya Medical Research Institute, Kisumu, Kenya; 4 Liverpool School of Tropical Medicine, Liverpool, UK; 5 Centre for Public Health, Liverpool John Moores University, Liverpool, UK; 6 President's Malaria Initiative, Washington DC, USA; 7 Michigan State University, East Lansing, USA

## Abstract

**Background:**

Insecticide-treated bed nets (ITNs) reduce malaria transmission and are an important prevention tool. However, there are still information gaps on how the reduction in malaria transmission by ITNs affects parasite genetics population structure. This study examined the relationship between transmission reduction from ITN use and the population genetic diversity of *Plasmodium falciparum *in an area of high ITN coverage in western Kenya.

**Methods:**

Parasite genetic diversity was assessed by scoring eight single copy neutral multilocus microsatellite (MS) markers in samples collected from *P. falciparum-*infected children (< five years) before introduction of ITNs (1996, baseline, n = 69) and five years after intervention (2001, follow-up, n = 74).

**Results:**

There were no significant changes in overall high mixed infections and unbiased expected heterozygosity between baseline (%M_A _= 94% and H_e _= 0.75) and follow up (%M_A _= 95% and H_e _= 0.79) years. However, locus specific analysis detected significant differences for some individual loci between the two time points. Pfg377 loci, a gametocyte-specific MS marker showed significant increase in mixed infections and H_e _in the follow up survey (%M_A _= 53% and H_e _= 0.57) compared to the baseline (%M_A _= 30% and H_e _= 0.29). An opposite trend was observed in the erythrocyte binding protein (EBP) MS marker. There was moderate genetic differentiation at the Pfg377 and TAA60 loci (F_ST _= 0.117 and 0.137 respectively) between the baseline and post-ITN parasite populations. Further analysis revealed linkage disequilibrium (LD) of the microsatellites in the baseline (14 significant pair-wise tests and *I^S^_A _*= 0.016) that was broken in the follow up parasite population (6 significant pairs and *I^S^_A _*= 0.0003). The locus specific change in H_e_, the moderate population differentiation and break in LD between the baseline and follow up years suggest an underlying change in population sub-structure despite the stability in the overall genetic diversity and multiple infection levels.

**Conclusions:**

The results from this study suggest that although *P. falciparum *population maintained an overall stability in genetic diversity after five years of high ITN coverage, there was significant locus specific change associated with gametocytes, marking these for further investigation.

## Background

Malaria continues to be a major global public health burden, causing 250 million clinical cases and over 1 million deaths each year. Sub-Saharan Africa accounts for 90% of these cases [1]. To combat malaria, insecticide-treated bed nets (ITNs) have emerged as an efficacious and cost-effective malaria prevention tool. Several previous trials conducted in areas of different malaria transmission patterns have demonstrated that ITNs reduce *Plasmodium falciparum *malaria transmission by 70-90%. Most importantly, these trials have provided substantial evidence that use of ITNs significantly reduces all-cause mortality and malaria morbidity in children less than five years of age [2]. Additionally, ITNs have been associated with significant reduction in the adverse effects of malaria during pregnancy [3]. The remarkable effectiveness of ITNs has led to an up-scaling of their use in malaria endemic regions in conjunction with other control and prevention measures [4]. Recently, the World Health Organization (WHO) reported that in countries where ITNs have been effectively scaled up, substantial reductions in malaria cases and deaths have occurred [1].

Insecticide-treated bed nets work by killing mosquitoes on contact and also by repelling or deterring the vectors from entering houses, thereby reducing malaria transmission [5]. Thus, the use of ITNs or increased distribution of ITNs not only affects the mosquito populations but also changes the dynamics of parasite dispersion in both human hosts and mosquito vectors, which could in turn modify vector-parasite-host interactions, ultimately affecting parasite populations. Several studies have shown that significant suppression of mosquito populations, changes in species distribution and vector behaviour, and changes in population genetic structure and susceptibility of mosquitoes to insecticides are associated with community-based ITNs intervention [6-9]. However, there are still information gaps on how the reduction in malaria transmission by ITNs affects parasite population genetic structure although there were a few earlier studies that reported no change in the proportion of multiple infections after transmission reduction by use of ITNs and curtains [10,11].

Human *Plasmodium *parasites undergo asexual multiplication in the human host and obligate sexual reproduction in the mosquito vector, each stage shaping the parasite population genetic structure. Although the asexual multiplication by haploid parasites in humans is clonal, polymorphism can arise from insertion/deletion of tandem repeats through slippage in the parasite DNA sequences or natural mutations from various pressures in the host-parasite relationship [12-14]. On the other hand, transmission of malaria parasites from human to mosquito, which is solely accomplished by a small number of infective male and female gametocytes generated in humans, creates an opportunity for the generation of parasite diversity and the emergence of novel genetic traits [15]. The parasite sexual reproduction stage in mosquitoes allows for recombination and re-assortment of genetic material between genomes of gametes to form diploid zygotes during the development of oocysts. The degree of inbreeding or outcrossing in mosquitoes influences the number of clones that are infective to human [16]. Numerous factors including host responses can indirectly influence competitive advantages or suppression of specific parasite clones. However, the level of transmission intensity has a direct effect on the number of infected hosts and number of parasite clones per infected individual, which affects parasite population genetic structure in different endemic settings [17,18]. Therefore, it is important to evaluate whether, and how, the transmission reduction (by use of ITNs or other methods), particularly in high transmission areas, affects the parasite population including the extent of multiple infections, genetic diversity, genes involved in transmission, drug resistance and polymorphism of vaccine candidate genes.

The study of parasite population structure explores the extent of genetic diversity, allele frequency, genotype distribution and degree of genetic admixture among other measures using statistical methods [19]. Common statistical measurements include expected heterozygosity (H_e_) to test genetic variation, linkage disequilibrium (LD) to assess association of alleles between loci, and fixation index (F_ST_) to evaluate population differentiation [20-22]. Natural *Plasmodium *parasite populations display extensive genetic variability within species at different geographic locations and different transmission intensity levels, with no predominant overall structure. Some studies of *P. falciparum *population structure report that areas with intense malaria transmission have higher H_e _and higher rates of outcrossing and recombination which breaks LD, resulting in a more panmictic population structure [17,23,24]. Such settings allow faster emergence of novel genotypes reflected as multiple infections. The reverse is expected where transmission is lower with consequent lower H_e_, stronger LD, and higher degree of selfing, resulting in a more clonal parasite population structure [17,25,26]. Yet other studies in areas with high malaria transmission have observed strong LD and non-random distribution of specific genotypes, implying inbreeding may be more extensive than expected even in areas with perennial transmission [27,28]. Although the conflicting results generated from different geographic regions could be partially due to the differences in genetic markers used, methods for estimation of allele frequencies or sampling of parasites at different life cycle stages, they underscore the need to study the relationship between the transmission intensity and the *P. falciparum *population genetic structure in same locality where changes in transmission intensity can be monitored. Interventions which impose reduction in transmission, such as ITNs at high coverage in malaria-holoendemic areas, provide a field experimental system for research on these questions. The information from such studies is also useful in designing molecular surveillance systems for ITNs and for other adjunct control programmes [29,30].

This study is part of a two-phase large-scale community-based trial conducted in western Kenya and designed to investigate the impact of ITNs on malaria morbidity and all cause mortality. The overall goal of these parasite population genetics studies is to assess the effects of transmission reduction by ITNs on the population genetic structure of *P. falciparum *parasites for a sustained period. The current study employed eight single copy multilocus neutral microsatellite markers to study the genetic diversity of *P. falciparum *using blood stage parasites collected from children less than five years old in the same area prior to and five years after the introduction of ITNs. Genetic diversity of the parasites between the baseline and post-ITNs was assessed by quantifying the extent of multiple infections, allele frequencies, H_e_, LD, and genetic differentiation.

## Methods

### Study area and study samples

This study was part of a two-phase community-based ITN trial conducted in Asembo area (Bondo District) of western Kenya, where malaria is holoendemic. The design and characteristics of the ITN trial have been detailed elsewhere [31-33]. Briefly, biannual population censuses and annual cross-sectional surveys were conducted in 60 villages in the 200 km^2 ^trial area during the rainy season of March to May between 1996 and 2001 to determine the effects of ITNs on malaria morbidity and all cause mortality in children below five years of age. During each cross sectional survey, blood samples were collected from children and parasitological, clinical, and demographic information were documented. Entomological monitoring of *Anopheles *density and sporozoite infection rates were conducted regularly throughout both phases of the trial. Approximately 98% of malaria infections were due to *P. falciparum *in the trial area. Before the ITN trial, entomological inoculation rate (EIR) was reported at 61.3 infective bites per person per year [34] and prevalence of parasitaemia was about 70% in children aged less than five years [31]. After the introduction of ITNs, it was estimated that ITNs reduced transmission by 90% at the early stage of the two-phase trials [6]. The EIR and prevalence of parasitaemia in May 2001 were 1.3 infective bites per person per year and about 34% in children less than five years old, respectively [33]. For the study presented here, the samples collected in the 1996 survey, just prior to ITN introduction for baseline measurements, and the samples from the 2001 follow-up survey, five years post-ITN intervention were utilized. Microscopically confirmed malaria positive blood samples were randomly selected in a subset of villages from the 1996 survey and were further matched by the villages in 2001 survey. Calculation of sample size was based on a hypothesized significant difference in overall heterozygosity (H_e_) between baseline and post-ITN parasite populations using a confidence level at 95% and margin of error at 5% [35]. Assuming a H_e _of 0.7 observed in high transmission areas [17] for baseline and a conservative change in H_e _to 0.5 observed in areas with medium transmission intensity [17] for ITN post intervention, a sample size of approximately 63 (+ 10-15%) was deemed adequate allowing for failure in laboratory testing. In total, a sampling frame of 69 samples from baseline and 74 samples from the post-ITN survey was achieved for this study. Parasite genomic DNA extraction from the stored blood samples was by the QIAamp DNA Mini Kit (Qiagen, CA, USA). Extracted DNA was stored at -20C until use. The study protocol was approved by the Ethical Review Committee of the Kenya Medical Research Institute, Nairobi, Kenya, the Institutional Review Board of Michigan State University, and the Institutional Review Board of Centers for Disease Control (CDC) Atlanta, Georgia.

### Microsatellite markers and genotyping

Eight single copy microsatellite markers (MS) were used for genotyping as listed in Table 1. Broadly, the microsatellites included: 1) five putatively neutral MS (Poly-α, PfPK2, ADL, TAA60, and TAA 109), 2) one MS (Pfg377) linked to the protein gene exclusively expressed during maturation of gametocytes transmission stage of parasites, and 3) two MS (EBP and P195) linked to the genes of asexual stage antigens under possible natural immune selection [36]. Among these MS markers, Poly-α, PfPK2 and Pfg377 are in the coding region [37]. The selected neutral MS markers were described earlier and have been used previously to study changes in genetic structure of *Plasmodium *parasites [17,27,37]. All amplifications were carried out using single reaction PCR with thermocycling conditions described elsewhere [38]. Fluorescent-labelled primers incorporated with either HEX (green) or FAM (Blue) dyes were used and the PCR products read on ABI (Applied Biosystems 3100) capillary sequencer. GeneMapper software (ABI) was used to automate measurement of microsatellite base-pair length and quantify peak height. Allele identity per locus was obtained directly after allocation of all peaks above 200 fluorescent units. Multiple alleles were quantified based on a method described previously [17] with identification of minor alleles set at peak heights of ≥30% of the predominant allele. Where amplification failed for any of the microsatellites, data was reported as missing and not used for haplotype definition.

**Table 1:** List of microsatellites and PCR primers used for microsatellites amplification

MS^§ ^name	MS primer sequence 5'-3'	MS linked genes	Acc. No.^¥ ^of linked genes	Chromosome
Poly-α^a^	AAAATATAGACGAACAGA	DNA polymerase alpha	L18785	4
	GAAATTATAACTCTACCA			
Pfg377^a^	GATCTCAACGGAAATTAT	Gametocyte specific protein	L04161	12
	TTATCCCTACGATTAACA			
PfPK2^a^	CTTTCATCGATACTACGA	Protein kinase	X63648	12
	AAAGAAGGAACAAGCAGA			
ADL ^b^	TACAGTGTTTATATATACCG	Fructose bisphosphate aldolase	M28881	14
	GCATAAATAATGTGAGCAGA			
EBP ^b^	TTCACAAGCCAAATATCA	Erythrocyte binding protein	M93397	13
	ATTCATAACTCCTTCAGA			
P195 ^b^	GAGTTAAAATATGTTACCT	Merozoite surface protein-1	X02919	9
	AAATATCACTATTCCTGT			
TAA60^a^	TAGTAACGATGTTGACAA	Hypothetical protein	AF010556	13
	AAAAAGGAGGATAAATACAT			
TAA109^a^	TAGGGAACATCATAAGGAT	Hypothetical protein	AF010508	6
	CCTATACCAAACATGCTAAA			

MS^§ ^depicts microsatellites. Acc. No.^¥ ^depicts Accession number. The letters in superscript depict the initial description of microsatellites as follows: (^a^) by [37], (^b^) by [36].

### Data analysis

Since the ITN intervention is one of long-term goals for malaria control programmes, the sampling strategy in the current study aimed at testing changes in genetic diversity of *P. falciparum *after an extended use of ITN while minimizing any spatial effects on the population. Potential biases were envisioned in the data analyses as allele frequencies might change over time in finite populations [39]. However, the prediction in this study was that the ITN-mediated transmission reduction would precipitate changes in the parasite population from panmictic (higher parasite diversity, expected for high transmission intensity) to a more clonal structure (lower parasite diversity, expected for low transmission intensity). Changes in genetic diversity of the parasites between the baseline and post-ITNs were, therefore, assessed by quantifying and comparing multiple infections, allele frequencies, H_e_, LD, and F_ST_.

Initial microsatellite data checking and data conversion was done using Excel Microsatellite Tool Kit, an add-in programme used to format raw microsatellite data in Microsoft Excel for consequent use in different genetics softwares [40]. Analysis of multiple infections was based on both predominant and minor alleles. Outcome measures for this analysis were the proportion of infections with more than one allele, the mean allele count for individual microsatellite loci, the overall proportion of infections with at least two alleles and the overall mean of the highest number of allele count detected by any of the microsatellites. The difference in proportions of infections with at least two alleles and the difference in the mean allele count between the baseline and post-intervention parasite populations were assessed using Pearson's chi-square and Wilcoxon tests respectively. Conversely, only the predominant allele defined by the highest peak in each electropherogram per locus was used to analyze the allele frequency and allele richness, H_e_, LD, and F_ST_. There are possible biases from using predominant allele techniques to determine allele frequency in multiple infections [41,42]. However, the method applied here was shown to be appropriate in previous studies using some of the microsatellite targets selected in this study [17,37,43,44].

The infinite allele model which is more appropriate for analysis of the complex patterns observed in microsatellite loci in *P. falciparum *was used for genetic analysis [45]. Allele number, frequency and richness per locus in each parasite population were obtained by FSTAT2 [46]. The measure for allele number, frequency and richness was to illustrate the composition and distribution of alleles in the population. Unbiased H_e _at each locus was calculated as H_e _= [*n*/(*n*-1)][1-Σ^n^_i = 1 _*p^2^_i _*] where *n *is the number of isolates sampled and *p_i _*is the frequency of the *i*th allele while sampling variance for H_e _(V_s(He)_) was calculated as V_s(He) _= 2(*n*-1)/*n*^3^[2(*n*-2)][Σ*pi*^3^-(Σ*pi*^2^)^2^] [17,22]. The difference in single locus heterozygosities between the two parasite populations was tested with standard error (SE) of the sampling variance by the method described earlier [22] using the z absolute values to obtain the *p*-values.

The prediction of the ITN-mediated decrease in transmission intensity and consequent reduction in genetic diversity was a possible increase in LD in the post-ITN parasite population. LD measures the degree of association between or among gene loci under the null hypothesis of no association [47]. As such,

individual pair-wise LDs for baseline and post-ITN parasite populations were first obtained respectively by the Fisher's exact test adapted for haploid data using ARLEQUINS 3.11 programme [48]. Multilocus LDs were further assessed from the overall index of association (*I^S^_A_*) using LIAN programme [49] for both baseline and post-ITNs respectively. The multilocus LD test measures non-random association among all loci. The test compares the variance of differences at LD with the variance expected in LD derived from 10,000 simulated data sets, H_0_: V_D _= V_e_. A significant LD was when the observed variance (V_D_) was greater than expected (V_e_). Index of association was expressed as *I^S^_A _*= (V_D_/V_e_-1)(r-1) where r is the number of loci tested. A 95% confidence limit was determined by Monte Carlo simulation.

In order to further assess genetic diversity before and after ITNs intervention, genetic differentiation was tested using the F_ST _estimator [50] implemented by FSTAT programme [46]. F_ST _is a comparison of the sum of genetic variability within and between populations based on allele frequency differences in populations. Interpretation of the F_ST _values at each locus was based on three categories defined earlier as no differentiation (0), low genetic differentiation (0 > 0.05), moderate differentiation (0.05-0.15) and great differentiation (0.15-0.25) [51].

When multiple tests were conducted, significant levels of *p*-value for the comparisons were adjusted using Bonferroni's correction [52].

## Results

Results in this study comprise outcomes from the analysis of the eight microsatellite loci on 69 and 74 *P. falciparum *positive samples from baseline and post-ITN surveys, respectively. Locus P195 had an overall amplification rate of 90% for the baseline parasite population. All other loci had amplification rates of 94-100%.

### Multiple infections

The extent of multiple infections was assessed based on the proportion of multiple alleles and mean allele counts (Table 2). Overall, more than 90% of samples from both baseline and post-ITN surveys had at least two or more alleles detected by any of the microsatellites targets. There was no significant difference in the overall proportions of multiple alleles between the baseline and post-ITN parasite populations. However, analysis of individual loci revealed that the Pfg377 marker had a significantly lower proportion of multiple alleles in the baseline (30%) compared to post-ITN parasite population (53%). All other loci showed similar proportions of multiple alleles in the two parasite populations.

**Table 2:** Comparison of proportion of multiple alleles and mean allele counts in the baseline and post-ITN parasite populations

Locus	Baseline population (n = 69)		Post-ITN population (n = 74)		*p-*value < 0.006*	
	
	% M_A_	M_AC _± SE	% M_A_	M_AC _± SE	% M_A_*	M_AC_*
Poly-α	64	2.12 ± 0.13	72	2.40 ± 0.14	0.289	0.264
Pfg377	30	1.36 ± 0.07	53	1.62 ± 0.09	**0.002**	0.008
PfPK2	85	2.94 ± 0.17	50	1.74 ± 0.11	0.021	**0.001**
ADL	45	1.62 ± 0.10	50	1.54 ± 0.07	0.479	0.918
EBP	57	1.75 ± 0.10	61	2.10 ± 0.14	0.566	0.128
P195	43	1.48 ± 0.08	48	1.62 ± 0.09	0.570	0.400
TAA60	51	1.77 ± 0.13	59	2.04 ± 0.12	0.044	0.046
TAA109	75	2.28 ± 0.14	76	2.23 ± 0.12	0.990	0.810

**Overall**	94.2†	3.4 ± 0.15‡	95.9 †	3.1 ± 0.12‡	0.830	0.178

% M_A _is the proportion of infections with more than one allele in each locus. M_AC _denotes the mean allele count and the respective standard error (SE) at each locus. The † marks the overall proportion of infections with at least two alleles while ‡ marks the overall mean of the highest number of allele count detected by any of the 8 microsatellites. The % M_A_* and M_AC_* show *p*-values for differences in proportion of multiple alleles and mean allele counts between the two parasite populations. Numbers highlighted in bold show significant differences at *p *< 0.0063 (with Bonferroni correction) for individual loci.

Results from the mean allele counts showed that PfPK2 had significantly higher allele counts in the baseline compared to that in the post-ITN survey. In contrast, Pfg377 had a relatively higher mean allele count (1.62 ± 0.09) in the post-ITN survey than in the baseline parasite population (1.36 ± 0.07) although it was not a statistically significant difference (*p *= 0.0076) after Bonferroni correction. There was no significant difference in the mean allele counts for all other microsatellite markers between the two parasite populations. Overall, mean allele count was similar in the baseline parasite population (3.4 ± 0.15) compared to the post-ITN parasite population (3.1 ± 0.12) (Table 2).

### Allele frequency and heterozygosity

**Figure 1:**
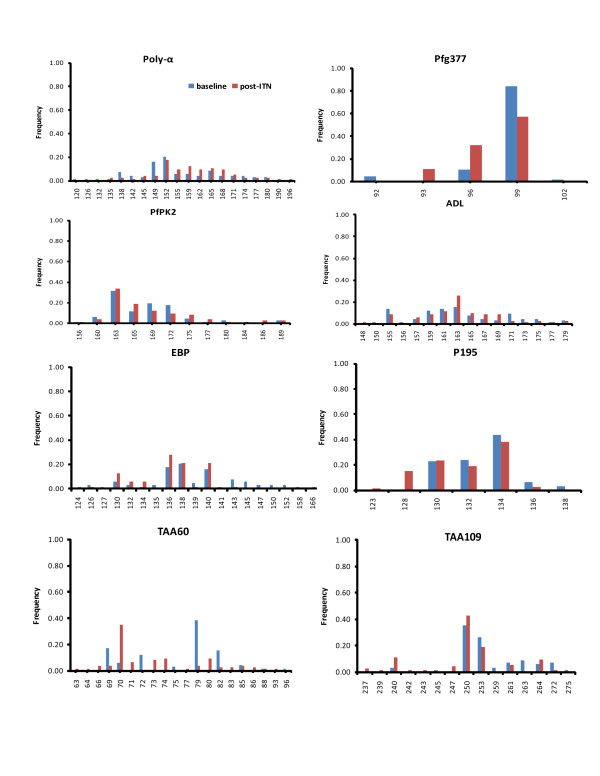
**Comparison of allele size and composition (base pairs, X-axis) and frequency distribution (Y-axis) of the eight individual microsatellites in the baseline (blue) and post-ITN (red) surveys**. The standardized Y-axis scale was used to depict the proportion of different alleles in each locus.

The allele size, number, frequency and richness for each MS shown in Figure 1 and Table 3 illustrated the composition and distribution of alleles. There were no differences between the allele number and richness as sample numbers used in the baseline and post-ITN surveys did not differ significantly for each locus. The allele number per locus ranged from a minimum of 4 (Pfg377) to maximum of 18 (Poly-α) in the baseline parasite population and from 3 (Pfg377) to 17 (Poly-α) in the post-ITN parasite population. This suggests marked variation between loci in the baseline and post-ITN surveys and was further tested by H_e_. Overall, H_e _was high and similar in the baseline (0.75 ± 0.072) and post-ITN parasite populations (0.79 ± 0.038). However, H_e _of individual loci showed extensive range in gene diversity (Table 3). Poly-α locus had the highest level of H_e _while Pfg377 locus showed the lowest in both parasite populations. Notably, Pfg377 locus had significantly lower H_e _in the baseline parasite population compared to the H_e _in the post-ITN survey (*p *= 0.044).

**Table 3:** Comparisons of genetic diversity between the baseline and post-ITN parasite populations

Locus	Baseline population		Post-ITN population		*p *< 0.05 (H_e_)*
		
	Allele Number (Allele Richness)	H_e _± SE	Allele Number (Allele Richness)	H_e _± SE	
Poly-α	18 (17.96)	0.91 ± 0.0159	17 (16.92)	0.92 ± 0.0243	0.7301
Pfg377	4 (3.99)	0.29 ± 0.0654	3 (3.00)	0.57 ± 0.0984	**0.0441**
PfPK2	10 (9.99)	0.82 ± 0.0235	12 (11.92)	0.83 ± 0.0497	0.8555
ADL	15 (14.99)	0.91 ± 0.0108	14 (13.96)	0.89 ± 0.0191	0.3620
EBP	17 (16.96)	0.90 ± 0.0168	11 (10.91)	0.82 ± 0.0193	**0.0017**
P195	5 (5.00)	0.71 ± 0.0310	6 (5.99)	0.75 ± 0.0241	0.3084
TAA60	9 (8.98)	0.79 ± 0.0323	17 (16.87)	0.85 ± 0.0322	0.1879
TAA109	10 (9.99)	0.79 ± 0.0301	11(10.91)	0.77 ± 0.0372	0.6760

Overall		0.75 ± 0.0720		0.79 ± 0.0380	0.9850

Comparisons of genetic diversity between the baseline and post-ITN parasite populations based on number of alleles at each locus, allele richness (in parenthesis), and the unbiased heterozygosity plus the standard error (H_e _± SE) [17,22]. (H_e_)* denotes the *p*-values for H_e _between baseline and post-ITN parasite populations.

### Linkage disequilibrium and genetic differentiation

Pair-wise LD for each individual MS locus for the baseline and post-ITN parasite populations was assessed. Of the 28 possible pair-wise tests LD was highly significant in a total of 14 pairs in the baseline parasite population while only 6 pairs were significant in the post-ITN population (*p *= 0.0018) as shown in Table 4. Specifically, in the baseline survey MS P195 locus (Chr9) and TAA60 (Chr9) had strong LD with loci located on different chromosomes including Poly-α (Chr4), Pfg377 (Chr12), PfPK2 (Chr12), EBP (Chr13) and ADL (Chr13). This association was however, reduced in the post-ITN population where 6 pairs showed significant LD. Only LD between P195 and ADL, and between TAA60 and EBP loci were maintained while the remaining four loci pairs were new associations in the post-ITN survey.

**Table 4:** Estimates of pair-wise linkage disequilibrium (LD) in the baseline and post-ITN parasite populations

Pair-wise *p*-values of LD (*p *< 0.0018) in the baseline and post-ITN parasite populations								
	**Poly-α**	**Pfg377**	**PfPK2**	**ADL**	**EBP**	**P195**	**TAA60**	**TAA109**

Poly-α		0.0050	**0.0004**	0.0048	**0.0006**	0.0092	0.1143	0.0310
Pfg377	0.0568		0.0546	0.0032	**0.0007**	0.0214	0.0157	0.0232
PfPK2	0.0037	0.3421		0.0471	0.0167	**0.0018**	0.0143	0.0658
ADL	0.0287	**0.0004**	**0.0013**		0.0867	**0.0001**	0.0026	0.0281
EBP	0.1107	0.0433	0.0043	0.0380		0.0255	0.0563	0.0256
P195	**0.0001**	**0.0001**	**0.0001**	0.0069	**0.0001**		**0.0007**	0.0223
TAA60	**0.0002**	0.4687	**0.0001**	**0.0002**	**0.0015**	**0.0001**		0.0102
TAA109	0.1151	0.0321	0.0050	**0.0001**	0.0095	**0.0001**	**0.0001**	

Comparison of pair-wise *p*-values of LD for MS in baseline (below diagonal) and post-ITN (above diagonal). Bold numbers indicate significant LD in the baseline and post-ITN parasite populations after Bonferroni correction.

The multilocus analysis showed significant LD in the baseline population with a variance in difference (V_D_) of 1.21 and variance expected (V_e_) of 1.10 (*p *= 0.01) compared to non-significant LD in the post-ITN population where V_D _and V_e _were 1.183 and 1.180 respectively (*p *= 0.57) as shown in Table 5. Consequently the index of association (*I^S^_A_*) was significant in the baseline survey (0.016) compared to the post-ITN survey (0.0003). The multilocus LD results coupled with the significant pair-wise LD observed in individual microsatellites suggest that the existing non-random association between the MS loci in the baseline was broken in the post-ITN parasite population.

**Table 5:** Estimates of multilocus linkage disequilibrium (LD) for baseline and post-ITN parasite populations

Test factor	Baseline population	Post-ITN population
V_D_	1.2089	1.1825
V_e_	1.0873	1.1802
*I^S^_A_*	0.0160	0.0003
**Testing (H_0_: V_D _= V_e_)**		
Var (V_D_)	0.0021	0.002
*p *< 0.05	**0.01**	**0.57**

Multilocus LD for all eight microsatellites markers examined before and after ITN intervention respectively. *P*-values shown are derived from Monte Carlo simulation methods for *I^S^_A _*showing levels of significant departure from 0 for each parasite population.

Results of the overall and locus specific genetic differentiation between the baseline and post-ITN surveys are shown in Table 6. Overall F_ST _was low at 0.027. In the single locus F_ST_, both Pfg377 and TAA60 markers showed moderate genetic differentiation (F_ST _= 0.117 and 0.137) respectively while all other remaining six loci had little differentiation. The results suggest that a moderately significant genetic variability at Pfg377 and TAA60 arose from differences between the two parasite populations while the majority of genetic variability remains within each population. Conversely, low genetic differentiation in the other six markers suggested much of the genetic variability resulted from within each parasite population.

**Table 6:** Genetic differentiation index (FST) between baseline and post-ITN parasite populations

Locus	F_ST_	Levels of Differentiation
Poly-α	0.003	Low
Pfg377	0.117	Moderate
PfPK2	-0.001	Low
ADL	0.000	Low
EBP	0.005	Low
P195	0.006	Low
TAA60	0.137	Moderate
TAA109	0.008	Low

Overall	0.027	Low

Genetic differentiation index (F_ST_) in each of the eight microsatellite loci between baseline and post-ITN parasite populations based on the null hypothesis that alleles are drawn from the same distribution in both parasite populations. The levels of differentiation were defined as low, moderate and great as described earlier [51].

## Discussion

The effect of five years of high coverage with ITNs on the genetic diversity of *P. falciparum *parasites was examined in this study. The overall proportion of mixed infections and heterozygosity were high at over 90% and 0.75 respectively both before and after ITN use with no significant reduction in these two parameters as well as the overall mean allele counts. This indicates an extensive multiplicity of circulating parasites in the area in spite of a dramatic decline in EIR post-ITN intervention. The results from this study are consistent with those of an earlier study conducted in areas with EIRs ranging from 0.4 to 31.8 in western Kenya, which also recorded over 80% mixed infections in both low and high malaria transmission areas [44]. This suggests the presence of a steady mix of circulating *Plasmodium *parasites in western Kenya despite reduction in EIRs.

The stable overall genetic diversity after dramatic reduction in transmission intensity observed in the current study was unexpected by the initial prediction. The counter-intuitive results suggest that other factors may be involved in offsetting the effect of transmission reduction on parasite genetic diversity and/or stabilization of the overall genetic diversity of malaria parasite. Indeed, several previous studies suggest that genetic diversity of malaria parasite measured by different markers could be shaped directly or indirectly by multiple factors such as seasonality, geographic scale, migration, disease severity, and host age and immunity [53-55] in addition to transmission intensity *per se *and natural selection. To minimize variation in host age, seasonality and geographic scale between baseline and post-ITN surveys, the current study sampled children less than five years of age, during similar transmission seasons and matched by villages in the two surveys. In addition, previous studies conducted in the ITN trial area showed that transmission reduction by use of ITNs changed humoral immunity in children and reduced childhood malaria morbidity and infant mortality resulting in overall decreased anti-malarial treatment [33,56,57]. The clinical and immunological outcomes after ITN intervention in the study area could potentially counteract the effect of transmission reduction on parasite genetic diversity and/or sustain overall higher genetic diversity although what mechanisms govern such a process within hosts is unknown. It is also possible that gene flow due to migration of mosquitoes and humans from surrounding non-ITN trial areas might contribute to the overall unchanged genetic diversity. However, the current study was not able to quantify the gene flow as the original ITN trial was not designed to include surrounding non-ITN areas after five years post-ITNs for comparison. Considering the parasite diversity could be influenced by multiple factors listed above, detection of change in parasite diversity within five years time window in the current study might not be sufficient. Currently, further studies on parasite population genetics are ongoing, which includes analysis of samples from approximately a decade later in the same ITN trial area and surrounding areas as well. Taken together, the unchanged overall genetic diversity observed in this study suggests a strong resilience of malaria parasite in response to dramatic transmission reduction after five years of sustained ITN use and possible involvement of other factors in stabilizing the overall parasite genetic diversity.

While the overall stability in the parasite genetic diversity show the transmission reduction by ITNs had insignificant impact on parasite population, locus specific changes suggest there were some differences in the parasite population sub-structure. For example, PfPK2 microsatellite marker showed a decrease in the mean allele counts in the post-ITN survey, while Pfg377 microsatellite locus showed a significant increase in the proportion of infections with more than one allele. There was also a decrease in genetic diversity (H_e_) in the EBP marker, but an increase at the Pfg377 locus. EBP MS locus flanks the functionally important erythrocyte binding protein gene expressed in the asexual stage of the life cycle and the gene may be under selection by human immune response [58]. It is possible that the decrease in H_e _for EBP MS marker observed in the post-ITN survey could reflect an indirect effect of ITNs on parasite genetic diversity but this will need further investigation. Likewise, PfPK2 MS which showed a decrease in mean allele counts flanks a putative protein kinase gene expressed in young trophozoite although the exact function is still not clear [59]. On the contrary, the MS located in the coding region of Pfg377 antigen gene specific for 'gametocyte-producing' parasites [60] showed an increased diversity in the post-ITN parasite population. Because there was either decrease or no significant changes in genetic diversity in other MS, the increase in genetic diversity for Pfg377 locus in the post-ITN parasites most likely reflected selection rather than genetic drift. Interestingly, the gametocyte carriage in the ITN trial area was significantly lower in the baseline survey (proportion 17% and mean density 12.4/ul) compared to the five years post-ITN survey (proportion 23%, mean density 41.2/ul) (CDC unpublished data). Taken together, this suggests that there is a possible relationship between the increased genetic diversity of Pfg377 and an increase in gametocyte carriage. The increased gametocytaemia and genetic diversity of Pfg377 locus could be an adaptive mechanism for transmission reduction to enhance the potential for parasite transmission to mosquitoes to maintain the life cycle for survival. This hypothesis requires testing to assess whether this gene has been a target of selection.

Consistent with the genetic diversity data described above, overall genetic differentiation between the baseline and post-ITN parasite populations was low, mainly arising from variations in the Pfg377 and TAA60 microsatellite markers. The differentiation observed in this study for Pfg377 and TAA60 were higher between the baseline and post-ITN surveys than that observed between three geographically different areas in western Kenya [44]. Yet F_ST _was much lower in our study at other remaining loci examined in the same study in western Kenya [44]. The lower F_ST _estimates at other loci observed in this study are expected since the samples were from the same area for baseline and post-ITN surveys. It is possible that the differentiation at TAA60 could represent random temporal effect/drift on the parasite population independent of transmission reduction. However, the differentiation observed at Pfg377 most likely resulted from the decreased transmission intensity by the use of ITNs rather than mere temporal effect since Pfg377 locus showed consistent increases in multiple infection and H_e _after ITN intervention.

The inter-relationship among LD, transmission intensity and genetic diversity of malaria parasites is complex and is still far from conclusive. The stronger LD observed in the baseline survey in our study area is consistent with the trend observed in previous studies conducted in the Democratic Republic of Congo, Zimbabwe and western Kenya lowland areas where malaria transmission is intense [17,27,28,44], suggesting the occurrence of high inbreeding in *P. falciparum *even in areas with intense and perennial transmission. It would be expected that decreasing transmission intensity by use of ITNs increases LD level based on a generalized assertion of higher LD in low transmission areas [17]. However, the results from five years post-ITN intervention in this study were surprising and interesting. After ITN intervention the pair-wise LDs were broken in 65% of physically unlinked loci (Table 4) and the multilocus LD was also not significant compared to the baseline survey (Table 5). The LD result from post-ITN parasite population could suggest that the overall parasite population became more panmictic after bed net intervention, which is contrary to earlier prediction of a more clonal structure after an ITN mediated transmission reduction. However, it is also possible that the unexpected decrease in LD in the post-ITN parasite population is partially masked by the increase in genetic diversity of Pfg377, the 'gametocyte specific' MS marker, but this will require further investigation.

## Conclusion

This study suggests that although the parasite population maintained an overall stability after ITN use, there were locus specific changes in the *P. falciparum *parasites contributing to the observed differentiation between the two parasite populations. Of note, the data on Pfg377 locus showed an increase in diversity ecologically associated with reduction in transmission intensity. Further studies are necessary to evaluate the usefulness of this marker and other gametocyte-specific gene markers as molecular tools for monitoring how changes in transmission reflect gametocyte population dynamics [61]. It is also important to monitor genetic structure of *P. falciparum *for extended periods, and in different geographic areas and in changing ITN coverage.

## Competing interests

The authors declare that they have no competing interests.

## Authors' contributions

WG carried out genotyping work and genetic data analysis, and wrote the manuscript. SK was responsible for sample processing and laboratory diagnosis for the ITN trial, and participated in the design of this study. WH, FTK, DT, PPH, JG and KL implemented and conducted the Kenya ITN trial including collection of samples and epidemiological data. WH, BN and KL were PIs for the two phase ITN trial. JG, EW, MH and LS participated in the design of this study. SC and JW assisted in sample size calculation, sample selection and data analysis. YPS was the PI and responsible for the design of this study, participated in data analysis, and wrote manuscript. All authors contributed to data interpretation, read and approved the final manuscript.
